# Dynamics of Lymphocyte Populations during *Trypanosoma cruzi* Infection: From Thymocyte Depletion to Differential Cell Expansion/Contraction in Peripheral Lymphoid Organs

**DOI:** 10.1155/2012/747185

**Published:** 2012-02-12

**Authors:** Alexandre Morrot, Juliana Barreto de Albuquerque, Luiz Ricardo Berbert, Carla Eponina de Carvalho Pinto, Juliana de Meis, Wilson Savino

**Affiliations:** ^1^Laboratory on Thymus Research, Oswaldo Cruz Institute, Oswaldo Cruz Foundation, 21045-900 Rio de Janeiro, RJ, Brazil; ^2^Department of Immunology, Microbiology Institute, Federal University of Rio de Janeiro, 21941-590 Rio de Janeiro, RJ, Brazil; ^3^Institute of Biology, Fluminense Federal University, 24020-140 Niterói, RJ, Brazil

## Abstract

The comprehension of the immune responses in infectious diseases is crucial for developing novel therapeutic strategies. Here, we review current findings on the dynamics of lymphocyte subpopulations following experimental acute infection by *Trypanosoma cruzi*, the causative agent of Chagas disease. In the thymus, although the negative selection process of the T-cell repertoire remains operational, there is a massive thymocyte depletion and abnormal release of immature CD4^+^CD8^+^ cells to peripheral lymphoid organs, where they acquire an activated phenotype similar to activated effector or memory T cells. These cells apparently bypassed the negative selection process, and some of them are potentially autoimmune. In infected animals, an atrophy of mesenteric lymph nodes is also observed, in contrast with the lymphocyte expansion in spleen and subcutaneous lymph nodes, illustrating a complex and organ specific dynamics of lymphocyte subpopulations. Accordingly, T- and B-cell activation is seen in subcutaneous lymph nodes and spleen, but not in mesenteric lymph nodes. Lastly, although the function of peripheral CD4^+^CD8^+^ T-cell population remains to be defined *in vivo*, their presence may contribute to the immunopathological events found in both murine and human Chagas disease.

## 1. Introduction

*Trypanosoma cruzi* is the causative agent of Chagas disease affecting more than 10 million people in Latin America. The parasite is transmitted by feces of infected insect vectors belonging to the family Reduviidae [1–3]. After infection, the initial acute phase of the disease progresses to an asymptomatic indeterminate period with virtually undetectable parasitemia and a strong humoral and cellular anti-*T. cruzi *responses. Up to several years after the initial infection, approximately 20 to 30% of all infected individuals develop a chronic inflammatory disease primarily affecting the heart [2–4]. Although different mechanisms have been proposed to trigger this pathology, there is a growing body of evidence that parasite persistence is associated with a chronic inflammatory response, which is the primary cause of Chagas disease [5, 6].

Experimental models of *T. cruzi* infection have been widely used to study various aspects of the infection. Acute infection in mice leads to strong activation of innate and adaptive immune response [7, 8]. In the course of infection, there is a fine change in the dynamics on the size of lymphocyte populations that contributes to regional specificities of the immune response in central and peripheral lymphoid organs: while there is an expansion in peripheral lymphoid organs such spleen and subcutaneous lymph nodes due to T and B cell polyclonal activation, we have observed an atrophy of the thymus and mesenteric lymph nodes in the infection [9]. The atrophy in such lymphoid organs seems to be associated with differences in lymphocyte proliferation and death [10–16]. 

In the thymus, we and others have identified that the severe thymic atrophy in acutely infected animals is mainly due to apoptotic depletion of CD4^+^CD8^+^ double-positive (DP) thymocytes undergoing differentiation [13, 14, 17–25]. However, regardless of thymic changes promoted by the acute *T. cruzi* infection, we have showed that the negative selection remains functional [26].

In a second vein, we have showed that, in contrast with the physiological condition, there is an abnormal release of DP thymocytes into the periphery during the course of the *T. cruzi* infection [12, 13] and that these cells acquire an activated phenotype similar to what is described for activated single-positive T cells [26].

The dynamics of cell populations in various lymphoid organs during the infection may reflect differential profiles of the adaptive immune response driving lymphocyte fluctuations in distinct compartments of the immune system. The impact of these alterations during the parasite infection is still unknown. Yet, it is conceivable that an abnormal release of nonselected thymocytes during acute infection may have an impact on the host immune responses against the parasite. In the present paper we will focus recent data concerning the thymic atrophy during the course of acute *T. cruzi* infection, as well as the dynamics of lymphocyte subsets in distinct secondary lymphoid tissues. 

## 2. Thymus Atrophy and the Negative Selection of Thymocytes in Chagas Disease

Several pathogens, including *T. cruzi*, cause thymic atrophy [17]. The mechanisms underlying this phenomenon are most likely linked to a particular pathogen-host relation established during infection. In the *T. cruzi* model, it has been shown that the inflammatory syndrome mediated by TNF-*α* during the acute phase of infection induces the activation of hypothalamus-pituitary-adrenal (HPA) axis with the consequent release of corticosterone [18, 27, 28]. The glucocorticoid rise is likely associated with profound effects on the changes observed in the thymuses of *T. cruzi*-infected mice, including the lymphoid and nonlymphoid compartments.

Yet, the thymic atrophy triggered by *T. cruzi* is more complex, with other host-derived molecules likely being involved. For example, thymic atrophy is not seen in *T. cruzi*-infected galectin-3 knockout mice [19]. On the other hand, the parasite-derived transsialidase is involved in the generation of intrathymic T-cell death [21, 25, 29].

The thymic microenvironmental changes seen after acute infection comprise the enhanced expression of extracellular matrix ligands and receptors, which correlates with a higher fibronectin-driven migration of DP thymocytes, and the abnormal raise of immature DP cells in lymph nodes [8, 12, 13, 30]. 

Recently, we have determined whether the changes of the thymic microenvironment seen following *T. cruzi* infection, would also lead to an altered intrathymic negative selection of the T-cell repertoire. It is largely established that interactions between TEC and thymocytes control the development of the thymic microenvironment and T-cell development. Previous studies have shown that the disruption of normal thymic architecture is known to affect the expression pattern of autoantigens by TEC and functionality of thymus [31–33]. Thymic medullary atrophy and decreased expression of Aire and TRAs have been reported in mouse models deficient in several genes involved in the NF*κ*B pathway, suggesting an important role of this pathway in the development of thymic medulla [34]. We showed that the expression of Aire and highly selective tissue-restricted antigens was readily detectable in whole thymus by real-time PCR analysis from infected mice, rather similar to controls. These data suggest that the expression of peripheral antigens in the infected thymuses is sufficient to modulate the tolerance induction by the negative selection process [26]. 

As the acute phase of infection progresses, the thymic atrophy becomes evident, as is the increase in numbers of apoptotic intrathymic DP cells, compared to their respective normal counterparts. Although this phenomenon may be a consequence of the changes observed in the organ, our data show that along the DP depletion there is sustained expression of Bim, proapoptotic factor essential for thymocyte negative selection. Finally, by using an OTII TCR transgenic system, we were able to demonstrate that the administration of the cognate OVA peptide in the acutely infected mice undergoing thymic atrophy can induce TCR-stimulation-induced apoptosis of semimature thymocytes. These data point out that negative selection operates normally during infection-promoted thymic atrophy, since the DP cells can be negatively selected in the infected thymus by antigen-induced depletion [26]. This supports previous work showing that intrathymic mature single-positive CD4^+^ or CD8^+^ T cells do not bear forbidden TCR genes as compared with their DP counterparts undergoing intrathymic differentiation [12]. 

## 3. Dynamics of Lymphocyte Populations in Peripheral Lymphoid Organs in Chagas Disease

**Figure 1:**
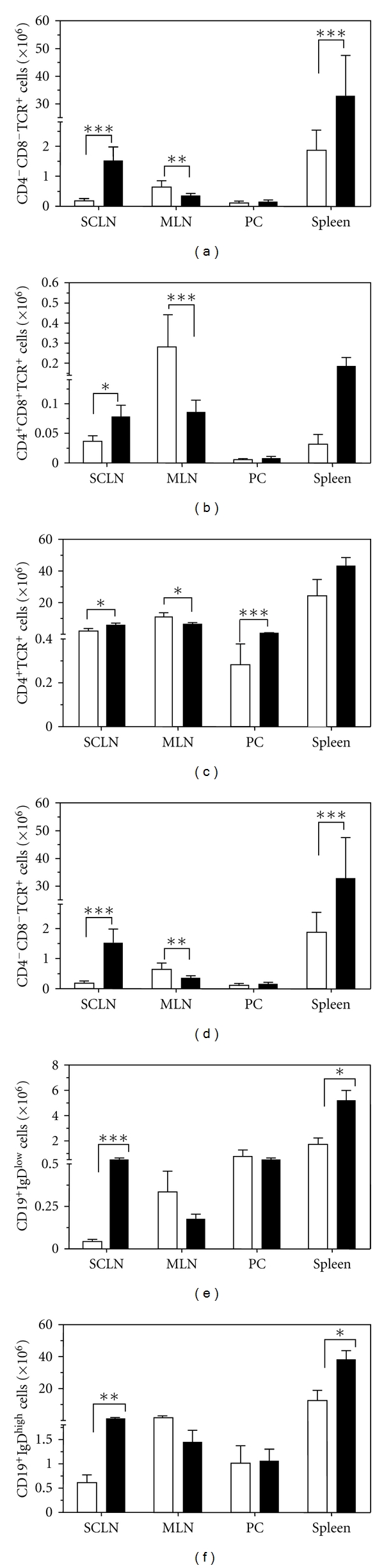
*Lymphocyte subsets in central and peripheral lymphoid organs from T. cruzi acutely infected mice*. Male BALB/c mice were infected intraperitoneally with 10² culture-derived trypomastigotes of *T. cruzi *(Tulahuén strain), and 14 days after infection the subcutaneous lymph nodes (SCLN), mesenteric lymph nodes (MLN), peritoneal cavity cells (PC), and spleen were harvested to perform flow cytometry. Erythrocytes were previously depleted in the spleen cell suspensions by treatment with Tris-buffered ammonium chloride. The total number of (a) double-negative CD4^−^CD8^−^ T cells, (b) double-positive CD4^+^CD8^+^ T cells, (c) single-positive CD4^+^ T cells, and (d) CD8^+^ T cells, (e) CD19^+^IgD^low^ B cells and (f) CD19^+^IgD^high^ B cells are indicated for each histogram. Values represent the mean and standard error. The infected group (*n* = 5–14) were compared to noninfected controls (*n* = 4–9) with *t*-test, using the program GraphPad Prism 5. Data were considered significant if *P* values were <0.05. *P < 0.05, **P < 0.01, ***P < 0.001.

The intrinsic balance among the lymphoid organs dictating the functional properties of the lymphocyte subsets are critical for establishing the immune response to *T. cruzi* infection [9]. Although the intrathymic checkpoints necessary to avoid the maturation of T cells expressing a forbidden T-cell receptor repertoire are present in the acute phase of murine Chagas disease, it has been shown that significant amounts of double-negative and double-positive thymocytes (Figure 1) are abnormally released from infected thymus to the periphery [12, 13, 35]. Considering that among thymus-derived CD4^+^CD8^+^ lymphocytes exhibit potentially autoimmune TCRs, we raised the hypothesis that they could be activated in peripheral lymphoid organs. This prompted us to evaluate in acutelyinfected mice whether those cells exhibited an activated profile similar to effector/memory single-positive T cells. The existence of this unconventional and rare (<5%) lymphocyte population in the periphery was explained as a premature release of DP cells from the thymus into the periphery, where their maturation into functionally competent single-positive cells continues [12, 35]. There is, however, considerable evidence of an increased frequency of peripheral CD4^+^CD8^+^ T cells during viral infections and during acute *T. cruzi* infection. For example, in human immunodeficiency virus or Epstein-Barr virus infections, the percentage of DP cells can increase to 20% of all circulating lymphocytes [36, 37]. This fluctuation is also present in the secondary lymph nodes as we demonstrated in the experimental model of Chagas disease, in which DP cell subset increases up to 16 times in subcutaneous lymph nodes [12, 13]. 

There is an increase in IL-2 production which has been associated with the hyperplasia in the subcutaneous lymph nodes during *T. cruzi* infection [9, 10]. Peripheral lymphoid organs play a role on the immunity against *T. cruzi* infection. For instance, it has been shown that splenocytes and mesenteric lymph nodes cells are involved in the host immune response since splenectomy or mesenteric lymph node excision prior to *T. cruzi* inoculum increases mice susceptibility to infection, as ascertained by the numbers of circulating parasites [9, 10]. 

The increase in cellularity of spleen and subcutaneous lymph node is a consequence of tissue T-/B-lymphocyte activation and expansion [9, 11, 38, 39]. Parasite-driven proteins seem to contribute to the polyclonal activation of lymphocytes in Chagas disease. In this regard, transsialidase seems to contribute to lymphocyte activation and cytokine production by interfering with interaction between dendritic cells and T lymphocytes [40, 41]. Other parasite-derived molecules such as racemase and *T. cruzi* DNA were proven to induce B-cell proliferation [42–44]. Although there are data showing that the polyclonal B-cell activation contributes to the pathological alterations seen in Chagas disease, evidence indicates that the majority of B cells are not parasite specific during early* T. cruzi* infection [41, 45–52].

Limited studies of B-cell dynamics among secondary organs during *T. cruzi* infection have been reported. CD19^+^IgD^low^ and CD19^+^IgD^high^ represent two functionally distinct B-cell subsets [53, 54]. CD19^+^IgD^low^ are located in the spleen and central lymphoid organs, at the marginal zones, acting as the first line responders to pathogens in the blood. These cells are able to migrate to germinal centers and participate in T-cell-dependent responses. CD19^+^IgD^high^ cells are found in the lymph and B-cell follicles of the spleen. We have shown that, 14 days after infection, both types of B cells are significantly expanded in the lymphoid organs such as spleen and subcutaneous lymph nodes (see Figures 1(e) and 1(f)). Since these B-cell subsets have distinct roles in the development of B-cell responses, related to their different localization, the increase of these two populations with polarized functions suggest a polyclonal B-cell activation during acute experimental Chagas. In agreement with this observation, previous reports suggests that CD5^+^ B cells are related to self-antigen immune response [55, 56].

In contrast to the hyperplasia seen in spleen and subcutaneous lymph nodes of infected mice, there is an atrophy of the mucosal associated lymphoid organs, which is related to a local decrease in IL-2 production, with a reduction of the total number of T/B lymphocytes [9]. One possible mechanism involved in IL-2 suppression in mesenteric lymph nodes could be associated with the differential distribution of regulatory T cells (Treg cells) among distinct lymphoid organs in infection, since IL-2 could be produced in normal levels and be sequestered by CD25^high^CD4^+^FOXP3^+^ cells, which have been demonstrated to be present in secondary lymphoid organs during infection [9, 57]. In this vein, the cytokine deprivation induced by Treg cells is one of the mechanisms that might be associated with apoptosis in T cells [58].

Considering that gut-associated lymphoid tissues drain antigens from the small intestine and that chronic infection may progress with damage to the digestive tract, mesenteric lymph nodes and Payer's patches might be related to gut pathologies seen in infected patients. Lymphocytes and dendritic cells that are primed in Payer's patches migrate to mesenteric lymph nodes through draining lymphatic vessels [9, 59]. Moreover, patients with digestive forms of the disease present high parasitemia and decreased T-/B-lymphocyte numbers in their blood [60, 61].

## 4. Concluding Remarks and Perspectives

**Figure 2:**
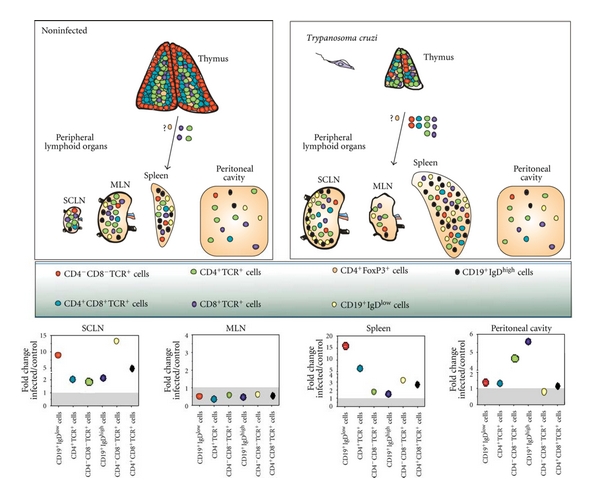
*Distinct pattern of lymphocyte fluctuations in central and peripheral lymphoid organs during acute Trypanosoma cruzi infection*. The upper panels depict thymus and peripheral lymphoid sites, in terms of T- and B-cell subsets. It is illustrated the thymic atrophy, simultaneously with an increase in mature and immature export of cells from the organ, as well as hyperplasia of spleen and subcutaneous lymph nodes that course in parallel with an atrophy of the mesenteric lymph nodes, whereas the peritoneal cavity remains rather unchanged (except for the significant rise in CD4^+^ and CD8^+^ T-cell subsets). Bottom panels reveal fold changes of each T- and B-lymphocyte subsets from acutely infected mice over the corresponding controls.

Our studies have revealed that the lymphocyte population size displayed significant fluctuations in the lymphoid tissues along the infection (Figure 2). Acute infection in mice leads to strong activation of innate and adaptive immune response. Splenomegaly and expansion in subcutaneous lymph nodes were reported, with persistent T- and B-cell polyclonal activation. Conversely, atrophy in mesenteric lymph nodes (MLN) and thymus was also observed in the infection.

Our data indicate that the key intrathymic checkpoints necessary to promote negative selection process of thymocytes are effective during acute chagasic thymic atrophy. Although the negative selection process is still functional in the acute phase, DP cells are prematurely released to the periphery. During the course of experimental infection, these peripheral DP cells acquire an activated phenotype that is correlated with the cardiac clinical form of human chronic Chagas disease. Therefore, although the function of this DP T-cell population remains to be defined* in vivo*, the presence of peripheral activated DP cells with potentially autoreactive TCR may contribute to the immunopathologic events found in both murine and human Chagas disease.

Overall we believe that the dynamics of T-cell expansion and contraction in persistent pathogen infections, herein exemplified by Chagas disease, is not limited to the fluctuations of the lymphocyte population size, but also the redistribution of lymphocyte subsets in the lymphoid tissues along the infection. Fluctuations in the lymphocyte cell population may reflect specific and coordinated responses to the parasite in the lymphoid organs.

The classical paradigm for T-cell dynamics suggests that the resolution of a primary infection is followed by the generation of a long-lived and stable pool of memory lymphocytes. Since the physical basis of the response is stochastic, very limited alteration in this repertoire is expected to occur due to alterations of the dynamics of the lymphoid tissues. Understanding the basis of stochasticity in lymphocyte fluctuations that occur secondary to *T. cruzi* infection will hopefully improve our knowledge on the regional influences upon the lymphocyte dynamics, and this will likely be useful for the development of protective immune responses necessary to control persistent infections.

## Abbreviations

*T. cruzi*: *Trypanosoma cruzi*;
DP T cells: CD4^+^CD8^+^ double-positive T cells
AIRE: Autoimmune regulator gene
TRAs: Tissue-restricted antigens
TCR: T-cell receptor
TEC: Thymic epithelial cells.
